# Guideline Concordance and Antimicrobial Stewardship in Community Pharmacy Management of Suspected Pediatric Acute Otitis Media: A Simulated Patient Study in Saudi Arabia

**DOI:** 10.3390/healthcare14193255

**Published:** 2026-10-01

**Authors:** Faris S. Alnezary, Nadia M. Aljuhani, Shuruq S. Almurashi, Reham A. Albuthayli, Adel Altarjami, Fahad Alzahrani, Haifa A. Fadil, Waad Alrohily, Masaad S. Almutairi

**Affiliations:** 1 Department of Pharmacy Practice, College of Pharmacy, Taibah University, Madinah 41477, Saudi Arabia; fnezary@taibahu.edu.sa (F.S.A.); nadia.m.aljuhani@gmail.com (N.M.A.); shurugsultan@gmail.com (S.S.A.); mreha2020@gmail.com (R.A.A.); adel.altarjami12@gmail.com (A.A.); fzahrani@taibahu.edu.sa (F.A.); hfadil@taibahu.edu.sa (H.A.F.); wrohily@taibahu.edu.sa (W.A.); 2 Department of Pharmacy Practice, College of Pharmacy, Qassim University, Qassim 51452, Saudi Arabia

**Keywords:** pediatric, acute otitis media, community pharmacy, simulated patient methodology, antimicrobial stewardship, clinical practice, practice guidelines, antibiotic dispensing

## Abstract

**Introduction:** Acute otitis media (AOM) is a common pediatric condition requiring careful assessment to distinguish children needing medical referral from those suitable for watchful waiting. Community pharmacists are often the first healthcare professionals consulted; however, evidence regarding the quality and guideline concordance of their AOM management remains limited. This study evaluated community pharmacists’ management of pediatric AOM across scenarios of varying clinical severity. **Methods:** A cross-sectional simulated patient study was conducted using three standardized pediatric AOM scenarios: a 5-month-old infant requiring referral, an 18-month-old child suitable for watchful waiting, and a 5-year-old child with multiple red flags requiring urgent referral. Trained simulated caregivers completed 203 community pharmacy visits. The primary outcome was scenario-specific guideline-concordant management. Secondary outcomes included referral, watchful waiting, antimicrobial recommendations, information gathering, counseling, and communication quality. Generalized estimating equation (GEE) logistic regression models accounted for clustering by simulated patient. **Results:** Guideline-concordant management was provided in 58.8% of Case 1 visits, 8.8% of Case 2 visits, and 58.2% of Case 3 visits. Referral was recommended in 60.3%, 47.1%, and 58.2% of visits, respectively, whereas watchful waiting was recommended in only 13.2% of the low-severity Case 2 visits. Antimicrobials were recommended in 7.4%, 22.1%, and 22.4% of visits, predominantly as topical antibiotics. Although pharmacists commonly assessed patient age and generally communicated professionally, assessment of symptoms, fever, ear discharge, medication use, allergies, and recurrent infections was inconsistent. Compared with Case 2, Cases 1 and 3 had substantially greater odds of guideline-concordant management. **Conclusions:** Community pharmacists’ management of suspected pediatric AOM varied considerably by clinical presentation. Major gaps were observed in watchful waiting, systematic clinical assessment, and antimicrobial stewardship. Standardized triage protocols and targeted professional training are needed to improve safe, guideline-concordant management.

## 1. Introduction

Acute otitis media (AOM) and upper respiratory infections (URIs) are leading pediatric illnesses globally, representing a severe clinical and public health burden [1,2]. These two conditions are deeply intertwined: viral URIs disrupt Eustachian tube function and delay bacterial clearance, precipitating secondary infections [3], while respiratory syncytial virus (RSV) significantly increases AOM risk [4]. Furthermore, core AOM bacterial pathogens like *Streptococcus pneumoniae* and *Haemophilus influenzae* are directly linked to upper respiratory colonization [5], a relationship further underscored by recent shifts in bacterial serotypes following pneumococcal conjugate vaccine adoption [6]. High incidence rates and severe complications are particularly pronounced in infants and young children, as well as in high-risk populations [7,8]. Globally, the direct and indirect economic impacts—driven by healthcare utilization and parental productivity losses—are profound [9,10].

Given this burden, guidelines advocate for risk-stratified management, reserving watchful waiting for low-severity cases while requiring immediate referral and antimicrobial therapy when red flags are present [11]. Despite these standards, inappropriate outpatient antibiotic use remains high, driven heavily by parental pressure and expectations for self-limiting viral conditions [12,13]. Cost–utility analyses indicate that parents frequently prioritize minimizing immediate sick days over the broader public health threat of antimicrobial resistance [14].

As accessible frontline providers, community pharmacists play a pivotal role in triaging minor ailments and safeguarding medication safety [15,16]. While integrating community pharmacies into primary care networks improves systemic efficiency [17], and expanded prescribing models can ease general practitioner workloads [18,19], translating this potential into real-world antimicrobial stewardship remains difficult. Pharmacists frequently face inappropriate patient demands for antimicrobials without clinical justification [20].

The non-prescription dispensing of antibiotics is a major driver of antimicrobial resistance [21,22]. Pharmacists often dispense antibiotics under pressure without adequate clinical assessment [20,21], a problem compounded by challenges such as complex pediatric dosing calculations [23]. Conversely, strict regulatory enforcement—such as the recent Saudi Ministry of Health mandates—has successfully curtailed non-prescribed antibiotic sales [24].

Evaluating real-world pharmacy performance is challenging because self-assessments suffer from recall bias and social acquiescence [25]. To neutralize the Hawthorne effect, the simulated patient (SP) methodology is a robust, gold-standard tool for assessing clinical practice [24,25], having been successfully deployed for childhood ailments such as fever and upper respiratory tract infection (URTI) [26]. Building upon this foundation, the primary aim of this study is to evaluate community pharmacists’ management of suspected pediatric AOM using an SP methodology across varying levels of clinical severity. Secondary objectives include assessing information gathering, counseling quality, communication efficacy, and inappropriate antimicrobial or over-the-counter recommendations.

## 2. Materials and Methods

### 2.1. Study Design and Setting

This study utilized a cross-sectional, simulated patient (SP) methodology—often referred to as a “mystery shopper” approach—to evaluate community pharmacists’ management of suspected pediatric AOM. The study was conducted in community pharmacies located in Madinah, Saudi Arabia, beginning in December 2025 and extending over a period of three months. The SP methodology was selected to minimize the Hawthorne effect and accurately capture routine clinical practice. The study population comprised pharmacies operating within Madinah. Based on recent registry data from the Saudi Commission for Health Specialties (SCFHS), there are approximately 350 eligible pharmacies in the city. Inclusion criteria encompassed all community pharmacies and hospital outpatient pharmacies accessible to the general public. Inpatient hospital pharmacies were excluded. To ensure the study adequately represented the city’s diverse pharmacy landscape, a random sampling strategy was employed within each geographic district of Madinah.

Ethics approval was obtained from the Institutional Research Ethics Committee of Taibah University (Reference number: COPTU-REC-55-20230224). To preserve the blinding of the SP visits and ensure authentic, real-world pharmacist responses, obtaining individual informed consent immediately prior to the consultation was not feasible, as it would introduce a significant Hawthorne effect. Therefore, in accordance with the approved ethical protocol, a general notification approach was utilized. The general notification and informed consent forms were provided to the management of the majority of regional pharmacy chains, hospital outpatient pharmacies, and select independent pharmacies. After the completely anonymous and non-punitive nature of the simulated patient methodology was explained, none of the administrations approached declined participation. The management subsequently distributed the forms to all pharmacists within their network. This ensured that pharmacists were ethically informed about the ongoing study while remaining blinded to the specific timing and identity of the simulated patients. Consequently, the 203 simulated visits were successfully conducted across a geographically and operationally representative cross-section of Madinah’s community pharmacies.

### 2.2. Simulated Patient Scenarios

Three standardized clinical scenarios, representing varying levels of clinical severity and required management strategies, were developed based on the risk-stratified diagnostic and management recommendations outlined in the American Academy of Pediatrics (AAP) Clinical Practice Guidelines for Acute Otitis Media [27]. The clinical characteristics, relevant history, and expected actions for each scenario are detailed in Table 1.

Prior to data collection, all three simulated patients (SPs) underwent a rigorous training program to ensure standardization and minimize observer bias. Training encompassed intensive role-playing sessions, script memorization, and the standardization of clinical presentations, body language, and responses to anticipated pharmacist inquiries, ensuring consistency across all visits. To test the feasibility of the scenarios and data collection instruments, a pilot phase comprising 15 preliminary visits (5 visits per SP) was conducted in community pharmacies outside the final study area. Data and experiences from the pilot phase were reviewed collaboratively to resolve discrepancies and refine the standardized opening prompts and Google Forms data collection sheet. Throughout the main study period, consistency and inter-rater reliability among the SPs were continuously maintained through bi-weekly debriefing sessions and joint case reviews to ensure uniform execution across all visits.

### 2.3. Study Procedure and Data Collection

During each visit, the SP entered the targeted community pharmacy and initiated a consultation with the available pharmacist using standardized opening prompts. SPs were trained to provide additional clinical information only if explicitly prompted by the pharmacist. Immediately after concluding the consultation and exiting the pharmacy, the SP recorded the interaction details using a structured, mobile-optimized data collection sheet to minimize recall bias. Captured variables included visit demographics (date, time, duration, pharmacist gender), the exact questions asked by the pharmacist, final management recommendations, and qualitative notes regarding potential unblinding. While the 203 total visits included multiple visits to some of the same participating pharmacies to cover different clinical scenarios, these repeat visits were strictly conducted during different working shifts. This strategy ensured that a different dispensing pharmacist was encountered during each visit, thereby preventing recognition of the simulated patients and preserving the blinding of the study. Because of this repeated sampling within the same facilities, generalized estimating equation (GEE) models were utilized to appropriately account for clustering at the pharmacy level.

### 2.4. Outcome Measures and Performance Rubric

The primary outcome was guideline-concordant management, defined a priori based on the expected clinical action for each specific scenario: immediate referral for Case 1, watchful waiting for Case 2, and urgent referral for Case 3.

Secondary outcomes assessed the quality of the consultation across several domains, including information gathering, counseling quality, and communication quality. Information gathering was evaluated based on whether the pharmacist asked critical history-taking questions regarding the patient’s age, presenting symptoms, symptom duration, fever, pain location, ear discharge, general condition, known allergies, and previously tried treatments. Counseling quality was assessed by the pharmacist’s ability to explain the reason for their recommendation, articulate the urgency of a referral when applicable, describe specific red-flag symptoms and timeframes for watchful waiting, and communicate appropriate over-the-counter (OTC) dosing, administration, and potential side effects. Finally, communication quality was scored based on the overall interaction style, which was categorized as empathetic and patient, professional and clear, rushed, dismissive, or uncertain. A manual appropriateness rating was also recorded for each visit, evaluating whether the final recommendation (e.g., referral, OTC only, or antibiotic recommendation) was clinically safe and appropriate for the given scenario.

### 2.5. Statistical Analysis

Descriptive statistics were used to summarize visit characteristics, information-gathering frequencies, counseling metrics, and recommendation patterns. Continuous variables are summarized using medians and interquartile ranges (IQRs), while categorical variables are presented as frequencies and percentages with 95% Wilson confidence intervals. To evaluate predictors of management outcomes while accounting for clustering at the pharmacy level (due to repeat visits across different working shifts), GEE logistic regression models with an exchangeable working correlation structure were employed. The models included a case scenario (with Case 2 as the reference scenario), observed pharmacist gender, and standardized visit duration as independent covariates. Case 2 (low-severity) was established as the reference scenario for comparative models assessing the odds of guideline-concordant management and referral recommendations. Statistical significance was defined as a two-sided *p*-value < 0.05. All advanced statistical analyses, including the GEE logistic regression models, were performed using R software version [4.3.2/4.6.1] (R Foundation for Statistical Computing, Vienna, Austria).

## 3. Results

### 3.1. Visit Characteristics

A total of 203 simulated patient (SP) visits were completed. The visits were evenly distributed across the three simulated scenarios, with 68 visits for Case 1 (5-month-old infant), 68 visits for Case 2 (18-month-old boy), and 67 visits for Case 3 (5-year-old girl). Male pharmacists accounted for most visits (n = 184; 90.6%), while female pharmacists accounted for 9.4% (n = 19) of the visits. The overall median visit duration was 1.0 min (interquartile range [IQR]: 1.0–2.0), ranging from a median of 1.0 min in Case 1 to 2.0 min in Case 3 (Table 2).

### 3.2. Guideline-Concordant Management and Clinical Outcomes

**Figure 1:**
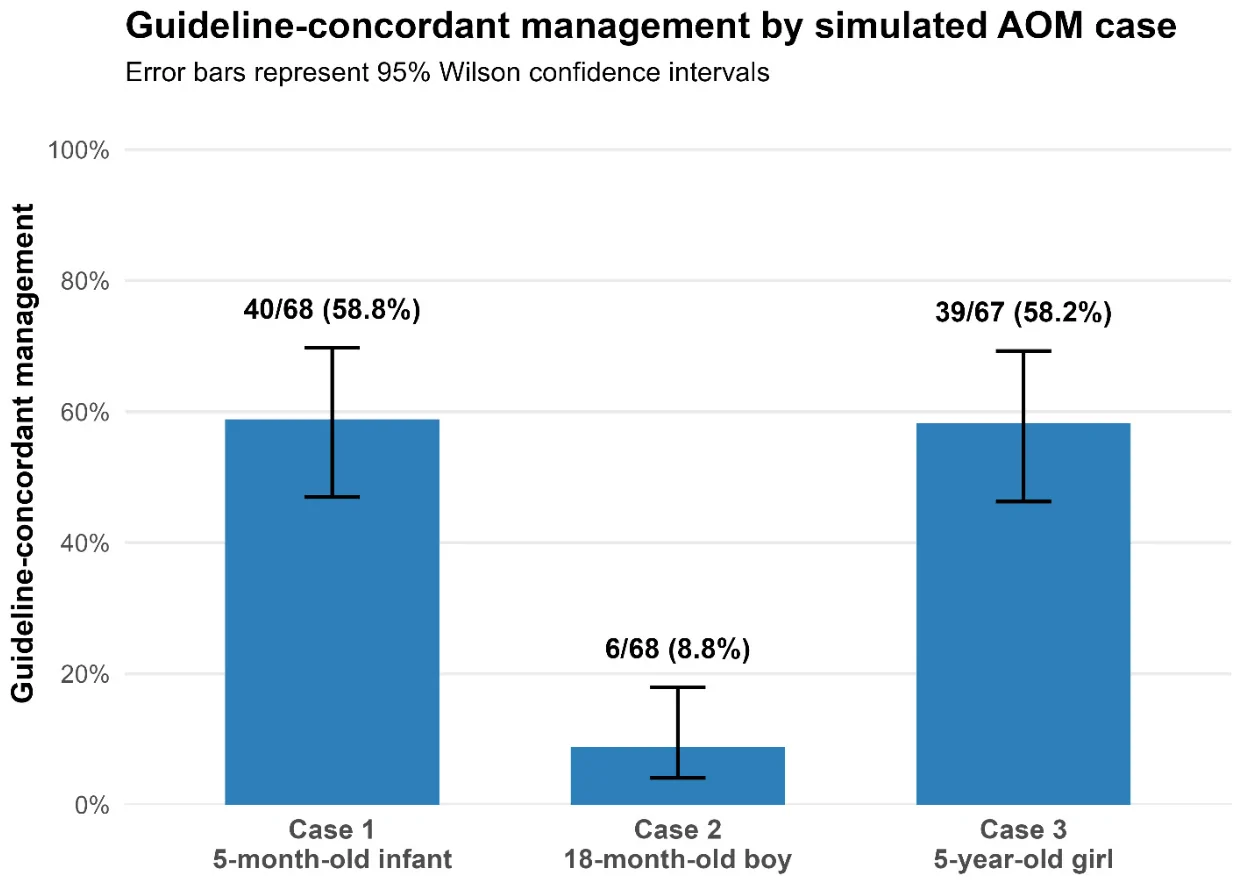
Guideline-concordant management by simulated case of suspected pediatric acute otitis media. Bars represent the proportion of simulated patient visits in which the pharmacist’s final recommendation was concordant with the expected clinical action for the scenario. Case 1 required physician referral because the patient was younger than 6 months; Case 2 was expected to receive watchful waiting with symptomatic relief; and Case 3 required urgent medical referral because of high fever, otorrhea, prolonged symptoms, and a history of recurrent AOM. Error bars represent 95% Wilson confidence intervals.

Guideline-concordant management was determined based on the expected clinical action for each scenario: referral for Case 1, watchful waiting for Case 2, and urgent referral for Case 3 (Table 3). Overall guideline concordance varied significantly by clinical presentation. Pharmacists provided concordant management in 58.8% (95% CI 47.0–69.7) of Case 1 visits and 58.2% (95% CI 46.3–69.3) of Case 3 visits, but in only 8.8% (95% CI 4.1–17.9) of Case 2 visits (Table 3, Figure 1).

**Figure 2:**
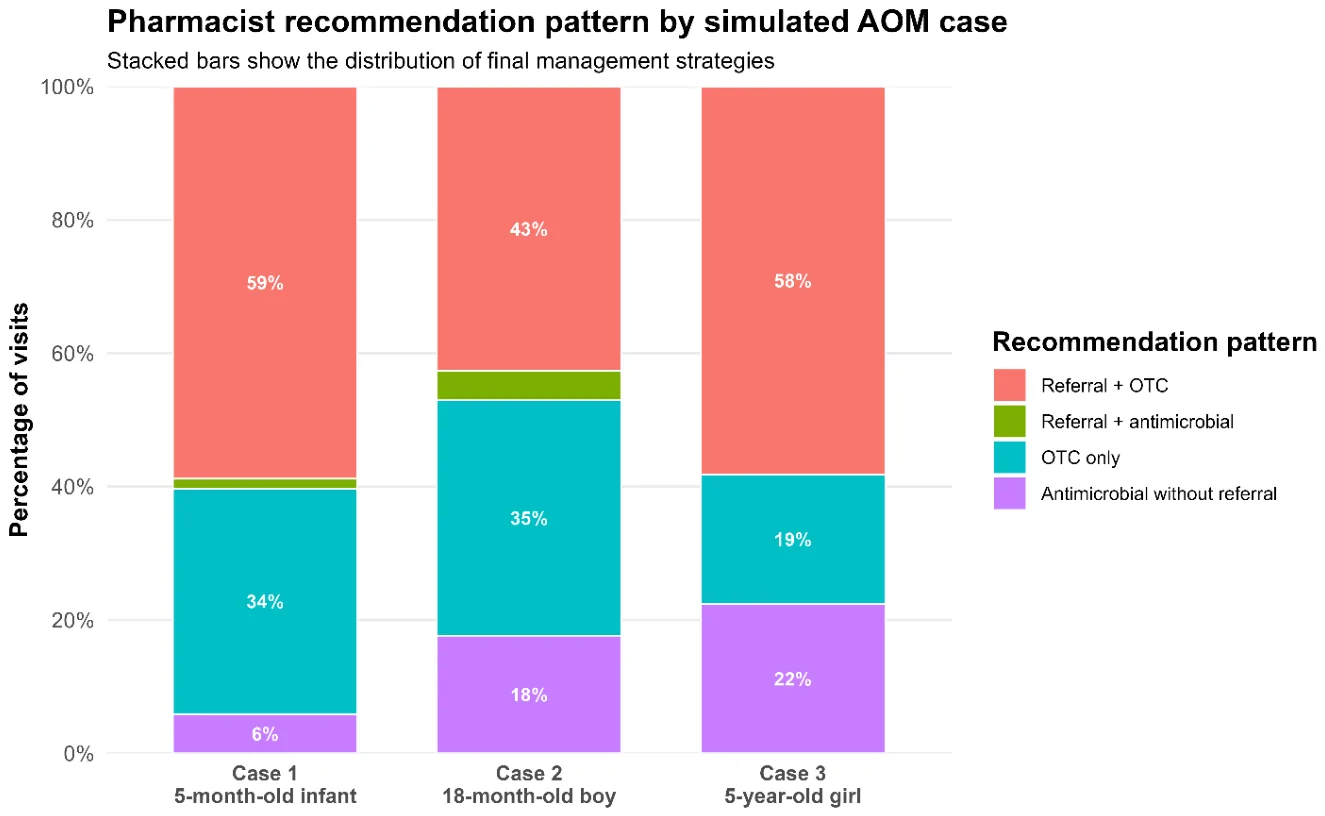
Pharmacist recommendation patterns by simulated case of suspected pediatric acute otitis media. Each stacked bar represents the distribution of final pharmacist management strategies across the three simulated cases. Recommendation patterns included referral only, referral plus OTC treatment, referral plus antimicrobial treatment, watchful waiting, watchful waiting plus OTC treatment, OTC treatment only, antimicrobial recommendation without referral, no intervention, no clear recommendation, and other management strategies.

Referrals were recommended in 60.3% of visits for Case 1, 47.1% for Case 2, and 58.2% for Case 3 (Table 3). Conversely, watchful waiting was recommended in 20.6% of Case 1 visits, 13.2% of Case 2 visits, and 4.5% of Case 3 visits. Final management strategies frequently involved a combination of referral and over-the-counter (OTC) treatments, which accounted for 59% of recommendations in Case 1 and 58% in Case 3, whereas OTC-only recommendations were observed in 34%, 35%, and 19% of Cases 1, 2, and 3, respectively (Figure 2, Supplementary Figure S2).

### 3.3. Antimicrobial and OTC Recommendations

**Figure 3:**
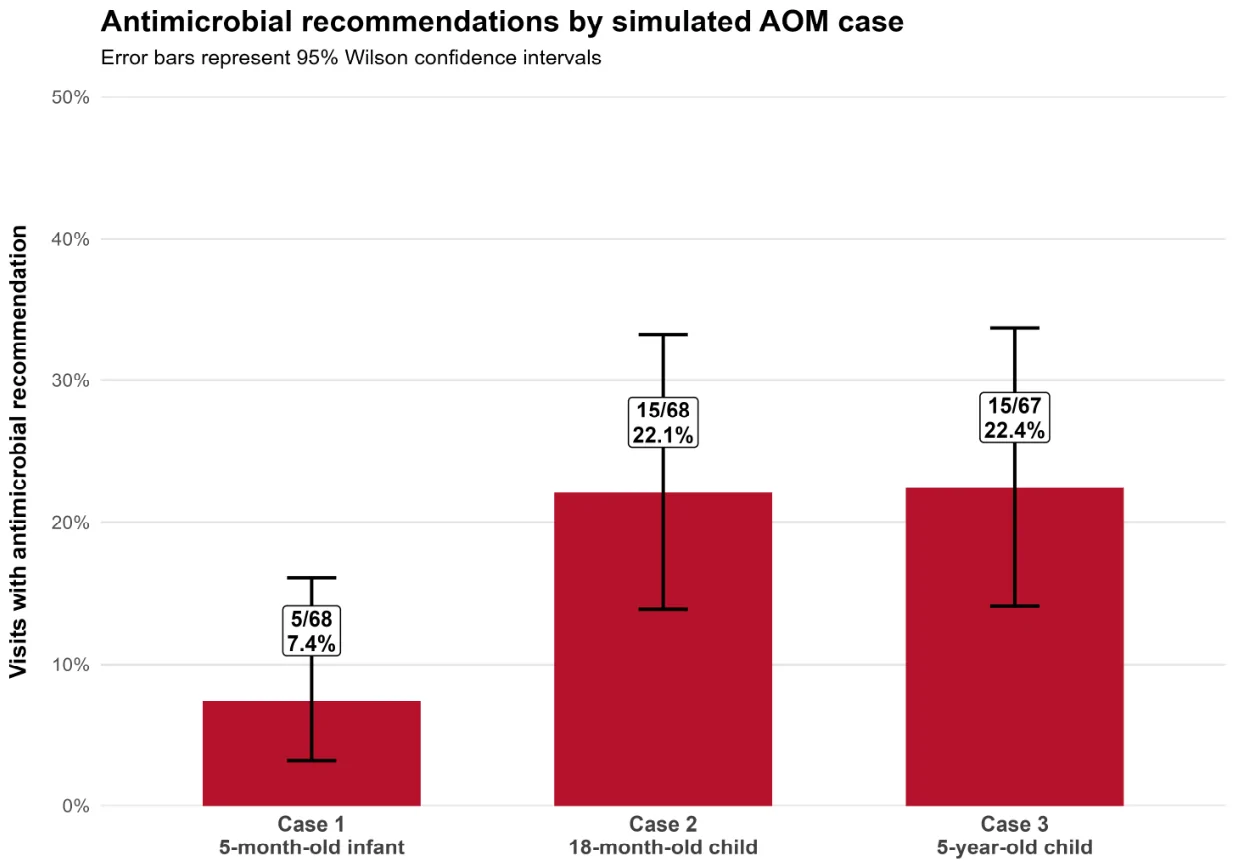
Antimicrobial recommendations by simulated case of suspected pediatric acute otitis media. Bars represent the proportion of simulated patient visits in which any antimicrobial was recommended, including oral antibiotics, topical antibiotics, or antifungal products. Error bars represent 95% Wilson confidence intervals. This figure highlights antimicrobial recommendation patterns across red-flag and low-severity AOM scenarios.

An antimicrobial was recommended in 7.4% (95% CI 3.2–16.1) of Case 1 visits, 22.1% (95% CI 13.8–33.3) of Case 2 visits, and 22.4% (95% CI 14.1–33.7) of Case 3 visits (Table 3, Figure 3). These recommendations were primarily driven by topical antibiotics, which were recommended in 5.9%, 20.6%, and 19.4% of visits for Cases 1, 2, and 3, respectively, whereas oral antibiotics were rarely recommended (1.5% to 6.0%) (Table 3).

An OTC product was recommended in 100% of the simulated visits across all three cases. OTC analgesics were recommended in 37% to 41% of visits, with paracetamol being more frequently recommended than ibuprofen in Cases 1 and 2, while ibuprofen was more common in Case 3. Anesthetic ear drops were recommended in 22% of Case 1 visits, 24% of Case 2 visits, and 36% of Case 3 visits.

### 3.4. Information Gathering, Counseling, and Communication

**Figure 4:**
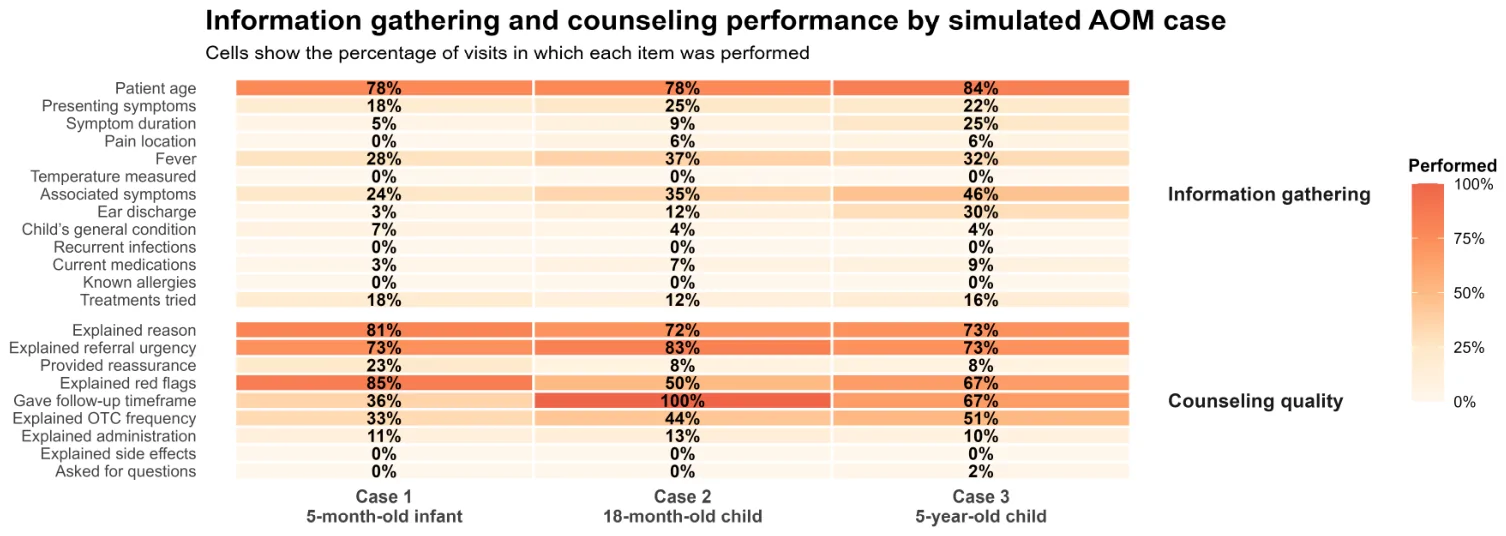
Information gathering and counseling performance by simulated case of suspected pediatric acute otitis media. Cells show the percentage of visits in which pharmacists performed each information-gathering or counseling item. Information-gathering items included assessment of age, presenting symptoms, symptom duration, fever, ear discharge, recurrent infections, medication history, allergies, and prior treatments. Counseling items included explaining the recommendation and referral urgency, providing reassurance, describing red-flag symptoms and the follow-up timeframe, explaining OTC administration details and side effects, and asking whether the caregiver had questions. Darker cells indicate higher performance.

Pharmacists frequently assessed patient age, performing this check in 77.9% to 83.6% of visits (Supplementary Table S1). However, other critical assessments were less consistent; pharmacists inquired about presenting symptoms in 17.6% to 25.0% of visits and asked about fever in 27.9% to 36.8% of visits (Supplementary Table S1, Figure 4). Information on symptom duration was gathered more frequently in Case 3 (25.4%) compared to Cases 1 and 2 (5.5% and 8.6%, respectively), and the frequency of questions regarding ear discharge was also highest in Case 3 (29.9%).

Pharmacist communication styles were most often rated as professional and clear (66.2% in Case 1, 69.1% in Case 2, and 82.1% in Case 3). Pharmacists explained the reason for their recommendation in the majority of visits (72.1% to 80.9%) and explained the urgency or importance of a referral when indicated (73.0% to 83.3%) (Supplementary Table S2). When watchful waiting was recommended, pharmacists described red-flag symptoms in 84.6% of Case 1 visits, 50.0% of Case 2 visits, and 66.7% of Case 3 visits and gave a timeframe for seeking medical care in 35.7%, 100%, and 66.7% of these visits, respectively (Supplementary Table S2, Supplementary Figure S1).

### 3.5. Predictors of Management Outcomes

GEE logistic regression models identified significant predictors of pharmacist management outcomes (Supplementary Table S3). Compared to Case 2, visits for Case 1 (odds ratio [OR] 12.99; 95% CI 4.77–35.37; *p* < 0.001) and Case 3 (OR 15.14; 95% CI 5.85–39.19; *p* < 0.001) had significantly higher odds of receiving guideline-concordant management. Pharmacist gender was also a significant predictor; male pharmacists had significantly lower odds of providing guideline-concordant management compared to female pharmacists (OR 0.26; 95% CI 0.11–0.60; *p* = 0.002) and significantly lower odds of recommending a referral (OR 0.14; 95% CI 0.03–0.58; *p* = 0.007). Visit duration was not significantly associated with guideline-concordant management (*p* = 0.646) or referral recommendations (*p* = 0.706).

### 3.6. Sensitivity Analysis

A sensitivity analysis comparing the manually recorded appropriateness rating with guideline concordance derived using rule-based criteria showed consistent trends across all three cases, confirming the reliability of the derived clinical classification (Supplementary Table S3, Supplementary Figure S3). Standardized models using the manual appropriateness rating corroborated the primary findings, demonstrating significantly higher odds of appropriate management for Cases 1 and 3 versus Case 2, and lower odds among male pharmacists.

## 4. Discussion

This simulated patient study identified substantial variation in community pharmacists’ management of suspected pediatric AOM. Under the prespecified criteria, guideline-concordant management was observed in approximately 58% of visits involving the 5-month-old infant and the 5-year-old child with multiple red flags, but in only 8.8% of visits involving the low-severity 18-month-old child. The main deficiency in Case 2 was the infrequent explicit recommendation of watchful waiting. Pharmacists therefore appeared more likely to recognize presentations requiring referral than to communicate a structured observation and safety-netting plan for mild symptoms.

The low concordance in Case 2 should be interpreted carefully. Watchful waiting does not mean withholding all care; it usually includes analgesia, monitoring, caregiver education, and instructions regarding when medical assessment is needed. Frequent recommendation of over-the-counter products does not itself indicate inappropriate practice. The more important concern was the absence of an explicit monitoring plan and complete safety-netting advice. Previous studies have similarly reported inconsistent implementation of watchful waiting in pediatric AOM [28,29]. However, because community pharmacists generally cannot confirm AOM by otoscopic examination, referral may also represent a clinically reasonable option. The low Case 2 concordance should therefore be understood as adherence to the study’s predefined rule-based rubric rather than proof that alternative management strategies were inherently unsafe, a finding further supported by our sensitivity analysis utilizing a broader manual appropriateness rating.

Referral was recommended in 60.3% of Case 1 visits and 58.2% of Case 3 visits, indicating moderate recognition of age-related and symptom-related red flags. Nevertheless, many pharmacists did not refer an infant younger than six months or a child with high fever, otorrhea, persistent pain, and recurrent episodes. This may delay diagnostic evaluation and supports the need for pharmacy-specific pediatric triage protocols.

Antimicrobials were recommended in 7.4% of Case 1 visits and approximately 22% of visits for Cases 2 and 3, with topical antibiotics accounting for most recommendations. The low frequency of oral antibiotic recommendations likely reflects the successful enforcement of Saudi regulations restricting non-prescription systemic antibiotic supply. Previous simulated patient studies have shown marked reductions in non-prescription antibiotic sales following such regulatory enforcement, although inappropriate dispensing has not been entirely eliminated [24,30]. However, evaluating these figures requires a precise clinical distinction regarding the appropriateness of topical agents. According to clinical guidelines, topical antibiotics offer no clinical benefit for uncomplicated AOM with an intact tympanic membrane, making their recommendation in the low-severity scenario (Case 2) clinically inappropriate. In contrast, for Case 3, the presence of otorrhea indicates a tympanic membrane perforation—a specific clinical presentation in which topical anti-infective drops are considered a guideline-supported therapy. Nevertheless, recommending or dispensing these topical antimicrobials without diagnostic confirmation remains a key stewardship and medication-safety concern. Verifying a tympanic membrane perforation necessitates an otoscopic examination, and providing these agents without a formal medical referral bypasses essential clinical safeguards. International evidence also shows that non-prescription antimicrobial supply may occur without adequate assessment and may increase following explicit client requests [21,31]. Patient demand, commercial pressure, and organizational barriers have been described elsewhere, but these factors were not measured in this study [32].

Assessment of the child’s age was the most consistent information-gathering behavior, occurring in approximately 78–84% of visits. In contrast, pharmacists infrequently assessed symptoms, duration, fever, ear discharge, general condition, medication use, previous treatment, allergies, or recurrent infection. No pharmacist asked about known allergies or recurrent AOM. These omissions are clinically important because safe pediatric triage depends on identifying red flags and ensuring that recommended treatments are appropriate. Similar gaps have been reported in Saudi Arabia, Palestine, and other Middle Eastern settings [33,34,35].

Communication was generally rated positively, and most pharmacists explained the reason for their recommendation. However, medication counseling remained incomplete. OTC administration instructions were rarely provided, dosing frequency was inconsistently explained, adverse effects were not discussed, and almost no pharmacist invited caregiver questions. A consultation may therefore appear professional while still omitting essential medication-safety and shared-decision-making elements. Comparable deficiencies have been described in community-pharmacy simulated patient studies [34,36].

Pharmacists’ male gender was associated with lower odds of guideline-concordant management and referral, though this finding must be interpreted as strictly exploratory. Because female pharmacists accounted for only 9.4% of the observed encounters (n = 19), this imbalance limits the statistical power of subgroup comparisons. More than 90% of visits involved male pharmacists, and information on experience, training, workload, and pharmacy characteristics was unavailable. Although gender-related professional dynamics and stereotypes may influence healthcare interactions, the available evidence does not explain the association observed in this study [37,38].

The main strength of this study was the simulated patient methodology, which assessed routine behavior without relying on self-report. Standardized scenarios also enabled comparison across levels of clinical severity. Limitations include the regional setting, inability to confirm AOM through otoscopic examination, absence of patient outcomes, small denominators for some counseling measures, and limited pharmacist- and pharmacy-level information.

These findings support practical interventions rather than education alone. Pediatric triage tools should prompt assessment of age, symptom duration, fever, ear discharge, general condition, allergies, recurrent infections, current medications, and prior treatment. When watchful waiting is considered, pharmacists should explain monitoring, analgesia, follow-up timing, and red-flag symptoms. Previous studies have identified barriers involving training, access to guidelines, reimbursement, and consultation space [35,39,40]. Community-pharmacy stewardship should therefore combine education with decision-support tools and clear referral pathways [41].

## 5. Conclusions

Community pharmacists’ management of suspected pediatric AOM varied substantially by clinical presentation. Pharmacists showed moderate recognition of referral scenarios, but important gaps remained in structured assessment, watchful-waiting communication, safety-netting, and medication counseling. Antimicrobial recommendations, particularly topical antibiotics without diagnostic confirmation, remain a stewardship concern. Standardized pediatric triage protocols, targeted training, and clear referral pathways are needed to improve the consistency and safety of community-pharmacy care.

## Figures and Tables

**Table 1:** Characteristics of simulated patient scenarios.

Case	Patient Profile	Presenter (SP Role)	Key Presenting Features	Relevant History	Designed Clinical Challenge & Expected Action
1	5-month-old male	Mother	Sudden onset of high-pitched crying (started last night). Pulling at the right ear and screaming when touched. Pain worsens when lying down.	Resolving cold/runny nose for the last 3 days. Refusing to breastfeed. Temperature: 37.3 °C; no known allergies.	To identify a high-risk red flag (patient <6 months with ear pain) and advise immediate physician referral.
2	18-month-old male	Older Sister	Intermittent ear discomfort for two days. Occasional ear tugging and mild irritability. Currently afebrile (36.9 °C), active, and playful.	Mild fever (38.0 °C) yesterday, which resolved on its own. No ear discharge or history of recurrent AOM. Eating and drinking normally.	To identify a low-severity case with no red flags and recommend a “watchful waiting” approach with symptomatic relief.
3	5-year-old female	Older Sister	Persistent right ear pain for three days. High fever (39 °C). Presence of ear discharge (otorrhea).	History of recurrent AOM (two episodes in the last six months). Minimal relief from paracetamol.	To identify multiple red flags (prolonged symptoms, high fever, otorrhea, recurrent infections) and advise urgent medical evaluation.

Note: Table content was derived directly from the standardized scenario rubric.

**Table 2:** Characteristics of simulated patient visits.

Characteristic	N	Overall ^1^	Case 1: 5-Month-Old Infant N = 68 ^1^	Case 2: 18-Month-Old Boy N = 68 ^1^	Case 3: 5-Year-Old Girl N = 67 ^1^
Simulated patient ID	203				
SP-1		66 (32.5%)	22 (32.4%)	22 (32.4%)	22 (32.8%)
SP-2		71 (35.0%)	24 (35.3%)	24 (35.3%)	23 (34.3%)
SP-3		66 (32.5%)	22 (32.4%)	22 (32.4%)	22 (32.8%)
Observed pharmacist gender	203				
Female		19 (9.4%)	9 (13.2%)	5 (7.4%)	5 (7.5%)
Male		184 (90.6%)	59 (86.8%)	63 (92.6%)	62 (92.5%)
Total visit duration, minutes	203	1.0 (1.0, 2.0)	1.0 (1.0, 2.0)	1.5 (1.0, 2.0)	2.0 (1.0, 2.0)

^1^ n (%); median (Q1, Q3).

**Table 3:** Pharmacist management outcomes by simulated case.

Values are n/N (%; 95% Wilson Confidence Interval)			
Outcome	Case 1: 5-Month-Old Infant	Case 2: 18-Month-Old Boy	Case 3: 5-Year-Old Girl
Guideline-concordant management	40/68 (58.8%; 95% CI 47–69.7)	6/68 (8.8%; 95% CI 4.1–17.9)	39/67 (58.2%; 95% CI 46.3–69.3)
Referral recommended	41/68 (60.3%; 95% CI 48.4–71.1)	32/68 (47.1%; 95% CI 35.7–58.8)	39/67 (58.2%; 95% CI 46.3–69.3)
Watchful waiting recommended	14/68 (20.6%; 95% CI 12.7–31.6)	9/68 (13.2%; 95% CI 7.1–23.3)	3/67 (4.5%; 95% CI 1.5–12.4)
Any antimicrobial recommended	5/68 (7.4%; 95% CI 3.2–16.1)	15/68 (22.1%; 95% CI 13.8–33.3)	15/67 (22.4%; 95% CI 14.1–33.7)
Oral antibiotic recommended	2/68 (2.9%; 95% CI 0.8–10.1)	1/68 (1.5%; 95% CI 0.3–7.9)	4/67 (6%; 95% CI 2.3–14.4)
Topical antibiotic recommended	4/68 (5.9%; 95% CI 2.3–14.2)	14/68 (20.6%; 95% CI 12.7–31.6)	13/67 (19.4%; 95% CI 11.7–30.4)

Guideline-concordant management was derived according to the expected clinical action for each scenario: referral for Case 1, watchful waiting for Case 2, and urgent referral for Case 3.

## Data Availability

Data will be provided upon request.

## Supplementary Materials

The following supporting information can be downloaded at: https://www.mdpi.com/article/10.3390/healthcare14193255/s1, Table S1: Pharmacist information gathering and clinical assessment by simulated case; Table S2: Counseling and communication quality by simulated case; Table S3: GEE logistic regression models for pharmacist management outcomes; Figure S1: Distribution of pharmacist practice-quality domain scores by simulated case of suspected pediatric acute otitis media; Figure S2: Clinical decision pathway from the simulated case of suspected pediatric acute otitis media to pharmacist recommendation pattern and guideline concordance; Figure S3: Comparison of manual appropriateness rating and derived guideline-concordant management by simulated case of suspected pediatric acute otitis media.
