# Supplementary material for: Guideline Concordance and Antimicrobial Stewardship in Community Pharmacy Management of Suspected Pediatric Acute Otitis Media: A Simulated Patient Study in Saudi Arabia
Source: Healthcare (Basel). 2026 Oct 1;14(19):3255. doi: 10.3390/healthcare14193255 (PMC13665284; doi:10.3390/healthcare14193255)
Supplement: Supplementary file 1 [file healthcare-14-03255-s001.zip › healthcare-4521094-supplementary.pdf]

Supplementary Table S1. Pharmacist information gathering and clinical assessment by simulated case

Values are n/N (%; 95% Wilson confidence interval)

Assessment item	Case 1: 5-month-old infant	Case 2: 18-month-old boy	Case 3: 5-year-old girl
Asked about patient age	53/68 (77.9%; 95% CI 66.7–86.2)	53/68 (77.9%; 95% CI 66.7–86.2)	56/67 (83.6%; 95% CI 72.9–90.6)
Asked about presenting symptoms	12/68 (17.6%; 95% CI 10.4–28.4)	17/68 (25%; 95% CI 16.2–36.4)	15/67 (22.4%; 95% CI 14.1–33.7)
Asked about symptom duration	3/55 (5.5%; 95% CI 1.9–14.9)	5/58 (8.6%; 95% CI 3.7–18.6)	17/67 (25.4%; 95% CI 16.5–36.9)
Asked about pain location	0/68 (0%; 95% CI 0–5.3)	4/67 (6%; 95% CI 2.3–14.4)	4/65 (6.2%; 95% CI 2.4–14.8)
Asked about fever	19/68 (27.9%; 95% CI 18.7–39.6)	25/68 (36.8%; 95% CI 26.3–48.6)	21/66 (31.8%; 95% CI 21.8–43.8)
Asked about temperature measurement	0/59 (0%; 95% CI 0–6.1)	0/57 (0%; 95% CI 0–6.3)	0/62 (0%; 95% CI 0–5.8)
Asked about associated symptoms	16/68 (23.5%; 95% CI 15–34.9)	24/68 (35.3%; 95% CI 25–47.2)	31/67 (46.3%; 95% CI 34.9–58.1)
Asked about ear discharge	2/68 (2.9%; 95% CI 0.8–10.1)	8/68 (11.8%; 95% CI 6.1–21.5)	20/67 (29.9%; 95% CI 20.2–41.7)

Supplementary Table S1. Pharmacist information gathering and clinical assessment by simulated case

Values are n/N (%; 95% Wilson confidence interval)

Assessment item	Case 1: 5-month-old infant	Case 2: 18-month-old boy	Case 3: 5-year-old girl
Asked about child's general condition	5/68 (7.4%; 95% CI 3.2–16.1)	3/68 (4.4%; 95% CI 1.5–12.2)	3/67 (4.5%; 95% CI 1.5–12.4)
Asked about recurrent infections	0/68 (0%; 95% CI 0–5.3)	0/68 (0%; 95% CI 0–5.3)	0/67 (0%; 95% CI 0–5.4)
Asked about current medications	2/68 (2.9%; 95% CI 0.8–10.1)	5/68 (7.4%; 95% CI 3.2–16.1)	6/67 (9%; 95% CI 4.2–18.2)
Asked about known allergies	0/68 (0%; 95% CI 0–5.3)	0/68 (0%; 95% CI 0–5.3)	0/67 (0%; 95% CI 0–5.4)
Asked about treatments tried at home	12/68 (17.6%; 95% CI 10.4–28.4)	8/68 (11.8%; 95% CI 6.1–21.5)	10/62 (16.1%; 95% CI 9–27.2)

Assessment items reflect history-taking and red-flag screening variables collected after each simulated patient visit.

Supplementary Table S2. Counseling and communication quality by simulated case

Values are n/N (%; 95% Wilson confidence interval)

Counseling/communication item	Case 1: 5-month-old infant	Case 2: 18-month-old boy	Case 3: 5-year-old girl
**Communication style**			
Dismissive	2/68 (2.9%; 95% CI 0.8–10.1)	3/68 (4.4%; 95% CI 1.5–12.2)	1/67 (1.5%; 95% CI 0.3–8)
Empathetic and patient	18/68 (26.5%; 95% CI 17.4–38)	11/68 (16.2%; 95% CI 9.3–26.7)	5/67 (7.5%; 95% CI 3.2–16.3)

Supplementary Table S2. Counseling and communication quality by simulated case

Values are n/N (%; 95% Wilson confidence interval)

Counseling/communication item	Case 1: 5-month-old infant	Case 2: 18-month-old boy	Case 3: 5-year-old girl
Professional and clear	45/68 (66.2%; 95% CI 54.3–76.3)	47/68 (69.1%; 95% CI 57.4–78.8)	55/67 (82.1%; 95% CI 71.3–89.4)
Rushed	4/68 (5.9%; 95% CI 2.3–14.2)	7/68 (10.3%; 95% CI 5.1–19.8)	5/67 (7.5%; 95% CI 3.2–16.3)
Uncertain or hesitant	2/68 (2.9%; 95% CI 0.8–10.1)	2/68 (2.9%; 95% CI 0.8–10.1)	1/67 (1.5%; 95% CI 0.3–8)
**Counseling quality**			
Asked if caregiver had questions	0/66 (0%; 95% CI 0–5.5)	0/65 (0%; 95% CI 0–5.6)	1/58 (1.7%; 95% CI 0.3–9.1)
Explained OTC administration	5/46 (10.9%; 95% CI 4.7–23)	7/52 (13.5%; 95% CI 6.7–25.3)	4/41 (9.8%; 95% CI 3.9–22.5)
Explained OTC frequency	15/46 (32.6%; 95% CI 20.9–47)	23/52 (44.2%; 95% CI 31.6–57.7)	21/41 (51.2%; 95% CI 36.5–65.7)
Explained OTC side effects	0/46 (0%; 95% CI 0–7.7)	0/52 (0%; 95% CI 0–6.9)	0/41 (0%; 95% CI 0–8.6)
Explained reason for recommendation	55/68 (80.9%; 95% CI 70–88.5)	49/68 (72.1%; 95% CI 60.4–81.3)	49/67 (73.1%; 95% CI 61.5–82.3)
Explained urgency/importance of referral	19/26 (73.1%; 95% CI 53.9–86.3)	20/24 (83.3%; 95% CI 64.1–93.3)	27/37 (73%; 95% CI 57–84.6)
Provided reassurance during referral	6/26 (23.1%; 95% CI 11–42.1)	2/24 (8.3%; 95% CI 2.3–25.8)	3/37 (8.1%; 95% CI 2.8–21.3)
Provided red-flag symptoms for watchful waiting	11/13 (84.6%; 95% CI 57.8–95.7)	4/8 (50%; 95% CI 21.5–78.5)	2/3 (66.7%; 95% CI 20.8–93.9)
Provided timeframe for seeking medical care	5/14 (35.7%; 95% CI 16.3–61.2)	8/8 (100%; 95% CI 67.6–100)	2/3 (66.7%; 95% CI 20.8–93.9)

Supplementary Table S2. Counseling and communication quality by simulated case

Values are n/N (%; 95% Wilson confidence interval)

Counseling/communication item	Case 1: 5-month-old infant	Case 2: 18-month-old boy	Case 3: 5-year-old girl

Denominators vary because some counseling items were applicable only when referral, watchful waiting, or OTC treatment was recommended.

Supplementary Table S3. GEE logistic regression models for pharmacist management outcomes

Odds ratios with 95% confidence intervals; models clustered by pharmacy

Predictor	Odds ratio (95% CI)	p-value
**Guideline-concordant management**		
Case 1: 5-month-old infant vs Case 2	12.99 (95% CI 4.77–35.37)	<0.001
Case 3: 5-year-old girl vs Case 2	15.14 (95% CI 5.85–39.19)	<0.001
Pharmacist gender: Male	0.26 (95% CI 0.11–0.60)	0.002
Total visit duration, per 1 SD increase	1.08 (95% CI 0.78–1.49)	0.646
**Manual appropriateness rating**		
Case 1: 5-month-old infant vs Case 2	7.17 (95% CI 2.78–18.50)	<0.001
Case 3: 5-year-old girl vs Case 2	11.29 (95% CI 4.43–28.79)	<0.001
Pharmacist gender: Male	0.34 (95% CI 0.14–0.83)	0.018
Total visit duration, per 1 SD increase	1.10 (95% CI 0.80–1.50)	0.566
**Referral recommended**		
Case 1: 5-month-old infant vs Case 2	1.40 (95% CI 0.69–2.84)	0.353
Case 3: 5-year-old girl vs Case 2	1.64 (95% CI 0.85–3.15)	0.136
Pharmacist gender: Male	0.14 (95% CI 0.03–0.58)	0.007
Total visit duration, per 1 SD increase	1.06 (95% CI 0.78–1.45)	0.706
**Any antimicrobial recommended**		
Case 1: 5-month-old infant vs Case 2	0.41 (95% CI 0.15–1.14)	0.087
Case 3: 5-year-old girl vs Case 2	0.87 (95% CI 0.41–1.86)	0.729

Supplementary Table S3. GEE logistic regression models for pharmacist management outcomes

Odds ratios with 95% confidence intervals; models clustered by pharmacy

Predictor	Odds ratio (95% CI)	p-value
Pharmacist gender: Male	2.49 (95% CI 0.49–12.73)	0.273
Total visit duration, per 1 SD increase	0.73 (95% CI 0.36–1.45)	0.365

Case 2 was used as the reference scenario. Visit duration was standardized; odds ratios for duration represent a one-standard-deviation (SD) increase.

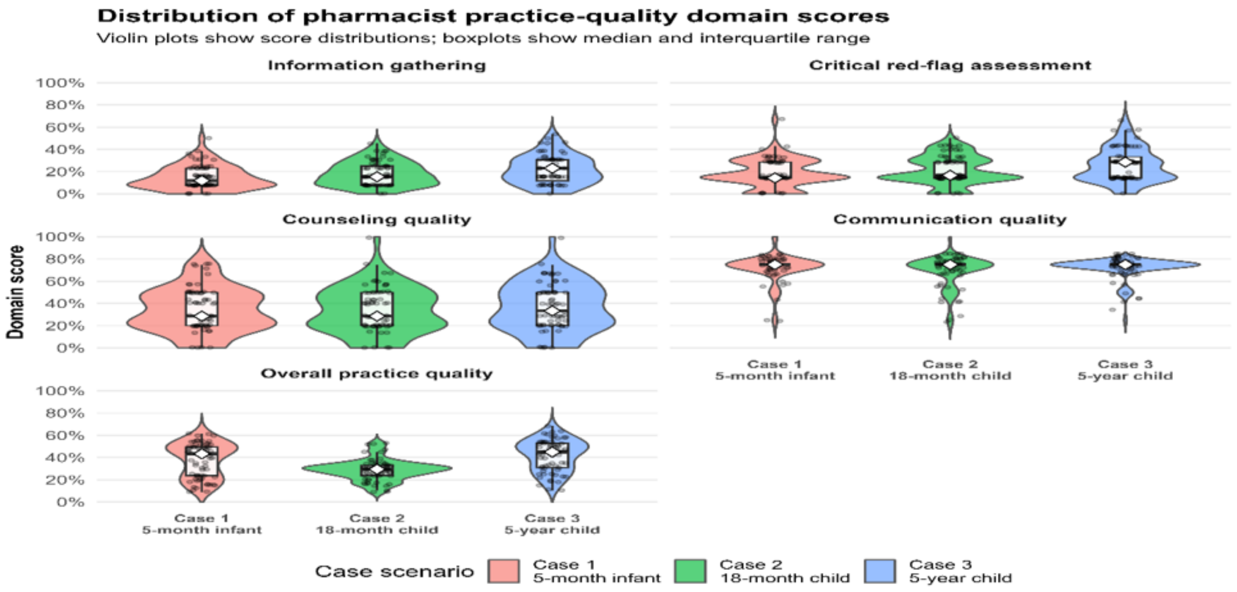

Supplementary Figure S1. Distribution of pharmacist practice-quality domain scores by simulated case of suspected pediatric acute otitis media. Scores range from 0 to 100, with higher values indicating better performance. Domains included information gathering, critical red-flag assessment, counseling quality, communication quality, and overall practice quality. Violin plots show score distributions, boxplots show medians and interquartile ranges, and individual points represent simulated patient visits.

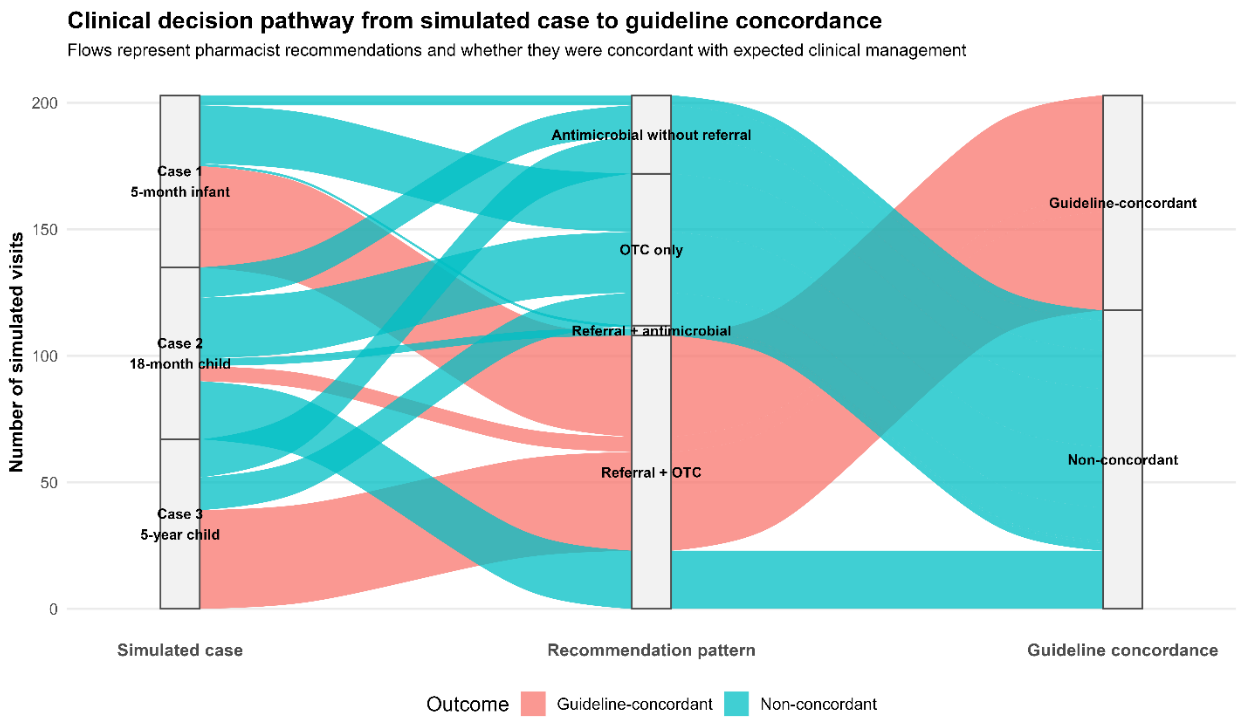

Supplementary Figure S2. Clinical decision pathway from the simulated case of suspected pediatric acute otitis media to pharmacist recommendation pattern and guideline concordance. Flows represent the number of simulated patient visits moving from each case scenario to the pharmacist's final recommendation pattern and final concordance classification. The figure illustrates how different clinical presentations translated into referral, watchful waiting, OTC treatment, antimicrobial recommendation, or non-concordant management.

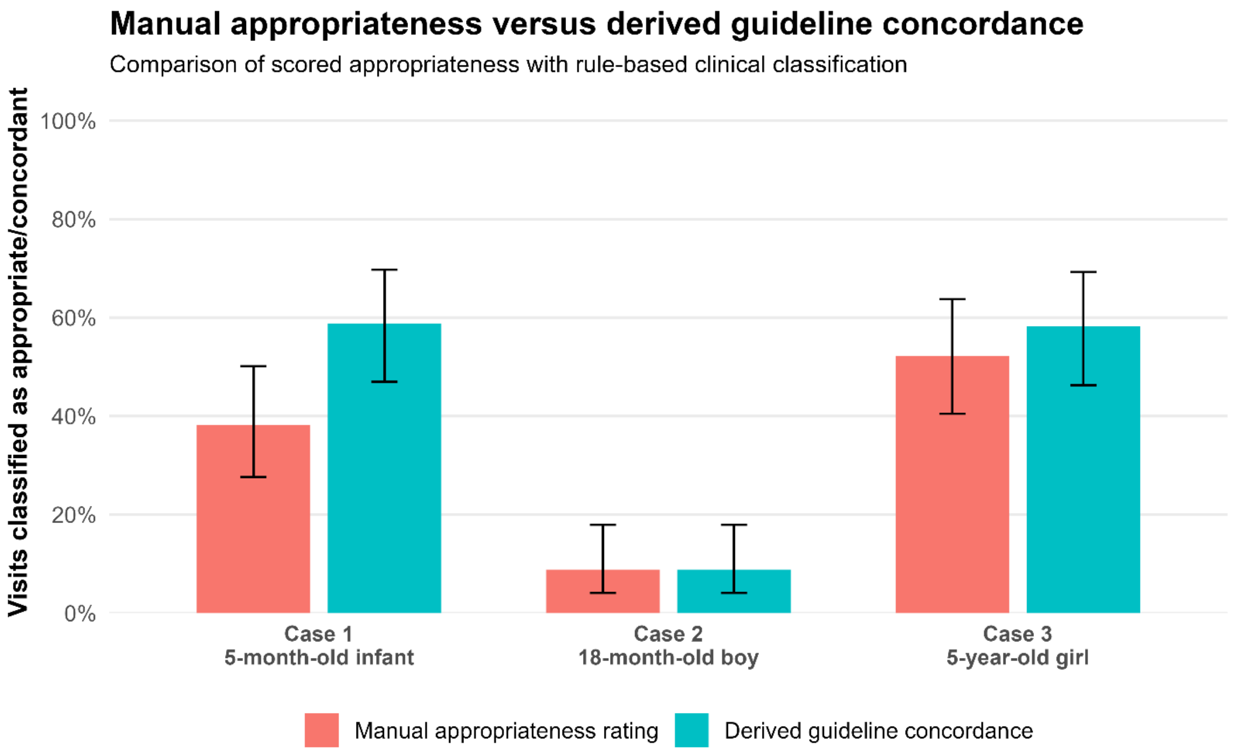

Supplementary Figure S3. Comparison of manual appropriateness rating and derived guideline-concordant management by simulated case of suspected pediatric acute otitis media. Bars

compare the proportion of visits classified as appropriate using the manually recorded appropriateness variable with the proportion classified as guideline-concordant using rule-based scenario-specific criteria. Error bars represent 95% Wilson confidence intervals. This sensitivity analysis evaluates consistency between manual scoring and the derived clinical outcome.
